# An osteoblast-like cell line derived from mice expressing FRET-based tension sensor reveals cellular tension increase during osteogenic differentiation

**DOI:** 10.1016/j.bbrep.2025.102131

**Published:** 2025-07-02

**Authors:** Junfeng Wang, Jeonghyun Kim, Eijiro Maeda, Takeo Matsumoto

**Affiliations:** Biomechanics Laboratory, Department of Mechanical Systems Engineering, Graduate School of Engineering, Nagoya University, Nagoya, Japan

**Keywords:** Osteoblast, Differentiation, Mineralization, Bone remodeling, FRET, Tension sensor

## Abstract

Mechanical stimuli significantly influence bone remodeling, although the detailed molecular mechanisms involving changes in intracellular tension during osteoblast differentiation remain unclear. The present study was performed to investigate the role of intracellular tension in osteogenic differentiation by utilizing a newly established osteoblast-like cell line. In this study, we established a novel osteoblast-like cell line derived from calvarial explants of transgenic mice ubiquitously expressing a Förster Resonance Energy Transfer (FRET)-based tension sensor, capable of real-time measurement of intracellular tension and mineralization. The established cell line FRET1-MC8 displayed superior proliferative ability compared to conventional MC3T3-E1 osteoblast-like cells, maintaining stable growth and mineralization capability through at least passage 65. Osteogenic medium (OM) significantly enhanced the expression of osteogenic differentiation markers *Col1a1* and *Spp1*, with clear mineralization observed as early as day 7 and extensive mineralization by day 14, comparable to MC3T3-E1 cells. Scratch experiments revealed increase in apparent contractility in OM-cultured cells, and subsequent quantitative analysis using acceptor photobleaching-based FRET efficiency confirmed significantly increased intracellular tension at day 3 of osteogenic induction. This elevation in intracellular tension coincided with increased *Spp1* expression, suggesting a critical role of tension in promoting osteogenic differentiation. The use of a FRET-based tension sensor enabled fast and direct monitoring of intracellular tension and is applicable to real-time analysis using live-cell FRET imaging. The established FRET1-MC8 cell line provides a powerful research tool for direct measurement of intracellular tension during osteogenic differentiation, thereby contributing to a better understanding of the mechanical regulation of bone remodeling.

## Highlights

• Novel osteoblastic cell line expressing a FRET-based tension sensor was established.
• The cells showed stable proliferation and mineralization capability over passage 65.
• Osteogenic induction increased intracellular tension and *Spp1* gene expression.
• FRET-based sensor may enable non-invasive real-time measurement of cellular tension.
• It is a useful tool for investigating mechanical regulation in bone remodeling.

## 1 Introduction

Bone is one of the most mechanically loaded tissues in the human body. When subjected to macroscopic external forces, the cells inside of bone, are subjected to mechanical stimuli such as shear stress, tension, and compression at the microscopic level [1]. Bone tissue maintains homeostasis through remodeling, which involves osteoclastic bone resorption and osteoblastic bone formation in response to these mechanical stimuli [2]. The lack of mechanical stimulation, such as physical inactivity, significantly induces bone loss [3], while mechanical loading through exercise can generally maintain or increase bone mass [4]. Thus, mechanical stimuli play a critical role in regulating the bone metabolism, as either insufficient or excessive stimulation increases bone remodeling activity [2].

To understand detailed mechanisms of bone remodeling, extensive research has been conducted by applying various types of mechanical stimuli, such as tension, compression, and shear stress, to bone tissues and bone-derived cells. For example, repetitive compression promoted mineralization and increased elastic modulus of chick tibia explants [5]. Real-time microscopic observation of chick bone explants under tensile loading further revealed the enhancement of mineralization along the aligned collagen fibers [6]. At a cellular scale, cyclic compressive loading enhances osteoblast differentiation, potentially relying on the Wnt/β-catenin signaling pathway concerning its magnitude and duration [7]. Additionally, shear stress applied to two-dimensional cultures of the osteoblast-like cell line MC3T3-E1 induced increased expression of osteoblastic differentiation markers [8]. Centrifugal mechanical stimulation of rat bone marrow-derived osteoblast-like RBM cells at 4800 rpm promoted cell proliferation and early osteoblast differentiation [9].

However, it remains unclear how extracellular mechanical stimuli are converted and transmitted into intracellular signaling and how mechanical states of cells are involved in such process. Quantitative measurement of intracellular tension may hold the key to understanding these phenomena because changes in intracellular tension are directly associated with alterations in nuclear and cytoskeletal structures, thus influencing gene expression and signal transduction [10]. Indeed, cytoskeletal tension has been reported to influence various cellular functions, such as regulating the differentiation of human mesenchymal stem cells [11] and modulating *Mmp-1* gene expression in tenocytes [12].

To measure changes in intracellular tension, tension sensors based on Förster Resonance Energy Transfer (FRET) were developed to quantitatively measure changes in intracellular tension in cultured cells [[13], [14], [15]]. Furthermore, we successfully generated transgenic mice expressing FRET-based tension sensor ubiquitously, confirming its functionality through observable changes in FRET ratio upon mechanical stimulation in isolated cells from various types of tissues [16]. However, to date, no cell lines derived from such reporter mice constitutively expressing FRET-based tension sensor units have been established, which can be a powerful experimental model to examine the involvement of intracellular tension in important physiological phenomena including bone remodeling.

Therefore, in the present study, we established an osteoblast-like cell line derived from the calvaria of these FRET-expressing transgenic mice, capable of measuring changes in intracellular tension during mineralization.

## 2 Materials and methods

### 2.1 Cell isolation and subculture

Cell isolation was carried out according to a method adapted from the establishment protocol of the osteoblast-like cell line MC3T3-E1 [17]. Briefly, transgenic FRET mice (line 1 or line 2, described previously [16]) aged 24–48 h, were euthanized by cervical transection, and skull bones were carefully isolated using forceps and scissors. Since mice used in this study were newborns aged 24–48 h, it was not possible to determine their sex. Periosteum and cartilage were gently removed from the bones in phosphate-buffered saline (PBS). Subsequent procedures were conducted aseptically in a clean bench. Calvaria were briefly immersed in PBS containing 10 % v/v penicillin-streptomycin (Nacalai Tesque, Japan) for 5 s and then cut into small pieces (∼1 mm × 1 mm) using a sterile scalpel blade. Bone fragments were placed into normal medium (NM) consisting of alpha-minimum essential medium (Wako, Japan) supplemented with 10 % fetal bovine serum (Gibco, USA) and 1 % penicillin-streptomycin (Nacalai Tesque, Japan), gently mixed, and seeded onto several 60-mm culture dishes. Each dish was assigned a cell line number; for example, cells derived from line 1 or 2 FRET mouse skull bones cultured in dish 1 were designated as FRET1-MC1 or FRET2-MC1, respectively. All procedures performed on these animals were approved by the Animal Care Committee of Nagoya University Graduate School of Engineering (Nos. 19–2, 20–5, GS220011). All methods were performed in accordance with the ARRIVE guidelines [18] and complied with all relevant guidelines and regulations for animal experimentation at Nagoya University.

Cells were incubated for 6 days at 37 °C in a humidified atmosphere with 5 % CO_2_, followed by detachment using 0.05 % Trypsin (Gibco) and centrifugation. Cell counts were performed using an automated cell counter (Countess II, Thermo Fisher, USA), and cells were re-seeded at a density of 120 cells/mm^2^ [17]. Subculturing was continued every 3–4 days, adjusting cell seeding densities within a range of 50–120 cells/mm^2^ according to growth conditions. Cell proliferation rate *Z*_Pn_ at passage number *n* was calculated as *Z*_Pn_ = *K*_Pn_/*H*_Pn_, and cumulative proliferation rates *C*_Pn_ = *Z*_P1_ × *Z*_P2_ × ··· × *Z*_Pn_, where *H*_Pn_ represents the number of cells seeded and *K*_Pn_ denotes the number of cells harvested.

Cells exhibiting stable proliferation were selected for further experiments. Expression of the FRET tension sensor was verified using a confocal laser scanning microscope (LSM880, Carl Zeiss, Germany) equipped with a 63 × objective lens (N.A. = 1.4, Carl Zeiss). The donor fluorophore EGFP was excited with a 488 nm laser (2 % of 19.5 mW maximum output), and fluorescence emissions from EGFP (495–550 nm) and the acceptor mCherry (580–624 nm) were collected. Phase-contrast images of subcultured cells were acquired using an inverted microscope IX73 (Olympus, Japan) equipped with a 10 × objective lens (N.A. = 0.3, Olympus).

### 2.2 Osteogenic differentiation and mineralization induction culture

To confirm the osteogenic differentiation and calcification capability of cells, a subgroup of isolated cells was cultured using an osteogenic medium (OM) modified from a previously described method [19]. Specifically, OM was prepared by supplementing NM with 100 μg/mL ascorbic acid (Wako), 20 mM β-glycerophosphate (Sigma, USA), and 10 mM calcium chloride (Wako). Cells were seeded at a density of 200 cells/mm^2^ in 35-mm plastic dishes with NM and cultured for 24 h, after which the medium was replaced with OM. The cells were then further cultured for designated periods. Subsequently, quantitative real-time PCR analysis of osteogenic marker genes and Alizarin Red S staining for calcium deposition were performed.

### 2.3 Quantitative real-time PCR analysis of differentiation markers

Cells cultured in OM for 2 or 7 days were lysed with ISOGEN II (Nippon Gene, Japan) for RNA extraction using the PureLink RNA Mini Kit (Thermo Fisher). RNA concentration was measured with a NanoDrop One spectrophotometer (Thermo Fisher). Subsequently, cDNA was synthesized using the ReverTra Ace qPCR RT Master Mix with gDNA Remover (Toyobo, Japan) and a MiniAmp thermal cycler (Thermo Fisher). Quantitative real-time PCR was performed using PowerUp SYBR Green Master Mix (Thermo Fisher) in QuantStudio 1 Real-Time PCR system (ThermoFisher). The target genes included early osteogenic markers runt related transcription factor 2 (*Runx2*) (NM_001146038.3; Forward: CCTGAACTCTGCACCAAGTCCT, Reverse: TCATCTGGCTCAGATAGGAGGG), alkaline phosphatase (*Alpl*) (NM_007431.3; Forward: GCTGATCATTCCCACGTTTT, Reverse: ACCATATAGGATGGCCGTGA), and collagen type I alpha 1 (*Col1a1*) (NM_007742.4; Forward: CGTGCAATGCAATGAAGAAC; Reverse: TCCCTCGACTCCTACATCTTCT); a mid-stage marker Osteopontin (*Spp1*) (NM_001204203.1; Forward: CCCGGTGAAAGTGACTGATT; Reverse: GGCTTTCATTGGAATTGCTT); and a late-stage marker osteocalcin (*Bglap*) (NM_001032298.3; Forward: GCGCTCTGTCTCTCTGACCT; Reverse: CGCCGGAGTCTGTTCACTAC). Glyceraldehyde-3-phosphate dehydrogenase (*Gapdh*) (NM_001289726.1; Forward: TGTTCCTACCCCCAATGTGT; Reverse: GGTCCTCAGTGTAGCCCAAG) was used as the reference gene. The primer sets were originally designed using Primer3 software (http://primer3.ut.ee/) as shown above, and their specificity was validated using NCBI BLAST software (http://blast.ncbi.nlm.nih.gov/). The gene expression levels were analyzed using the ΔΔCt method [20] with QuantStudio Design & Analysis Software (Thermo Fisher). The ΔCt value was calculated by normalizing each target gene's Ct value to that of *Gapdh*. The ΔΔCt value was obtained by comparing the ΔCt of the experimental group with that of the control group. Relative gene expression was calculated as 2^(-ΔΔCt). Cells cultured in NM for 2 days were used as the control group.

### 2.4 Alizarin Red S staining

Cells cultured in NM or OM for 7, 10 and 14 days were fixed with formalin solution for 10 min, then stained with 1 % (w/w) Alizarin Red S (Wako) solution (diluted in distilled water) for 30 min to detect calcium deposition. After staining, samples were rinsed three times with distilled water, and images were captured.

### 2.5 Scratch experiment

To assess apparent changes in cellular tension under osteogenic differentiation, scratch experiments were conducted as previously described [21]. Cells were seeded at 200 cells/mm^2^ density in 60-mm plastic dishes with NM. After culture, scratches were manually created with a sterile scalpel under an inverted microscope IX73. The dishes were fixed onto the microscope stage, and the scratching procedure was recorded at 10 frames per second for 3 min using a CCD camera (DP73, Olympus) with a 4 × objective lens. Cell migration and contracting responses were analyzed from the captured video images with a software ImageJ (1.54f, NIH).

### 2.6 FRET measurements

Cells were seeded at 200 cells/mm^2^ on glass-bottom dishes in NM and cultured for one day. Subsequently, the medium was replaced with either NM or OM, and cells were cultured for an additional 1 or 3 days. Cells were then fixed with 10 % formalin. Intracellular tension was quantified using the acceptor photobleaching method [22] to measure FRET efficiency. It has been reported that formalin fixation minimally affects intracellular tension [23]. Images were captured using a confocal laser scanning microscope (Zeiss LSM880) with a 63 × objective lens (N.A. = 1.4). EGFP was excited using a weak 488 nm laser (0.5 % intensity, max output 19.5 mW), and baseline donor fluorescence (*I*_DPre_) was recorded. Subsequently, acceptor fluorescence was photobleached by applying a full-power 543 nm laser to the entire image, and donor fluorescence intensity after bleaching (*I*_DPost_) was recorded using a higher intensity 488 nm laser (5 %). FRET efficiency (*E*) was calculated using the formula: *E* = 1 - (*I*_DPre_/*I*_DPost_).

### 2.7 Statistical analysis

Quantitative real-time PCR analysis data are presented as mean ± standard error (SE), while other data are expressed as mean ± SD. Statistical significance between groups was evaluated using Student’s t-test, and the statistical significance level was set at *P* < 0.05.

## 3 Results

### 3.1 Expression of FRET tension sensor and proliferation characteristics

**Fig. 1:**
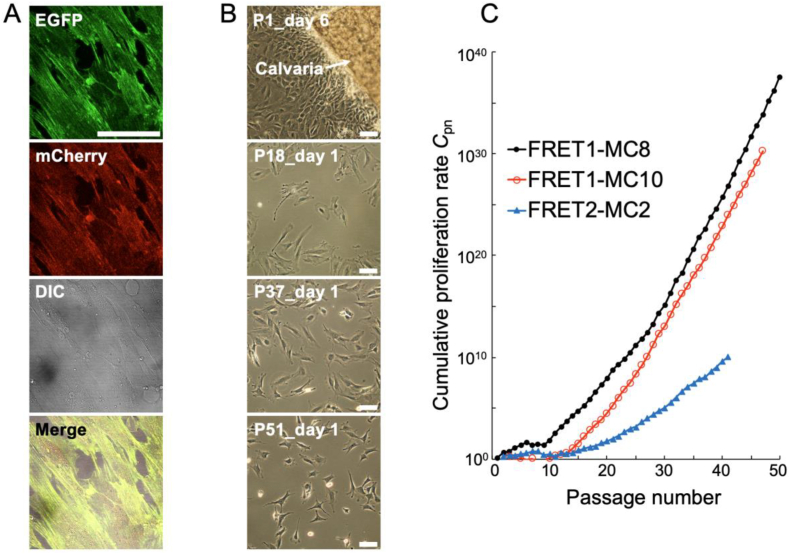
Expression of FRET tension sensor and proliferation characteristics. (A) Fluorescent images of FRET donor EGFP, acceptor mCherry along with differential interference contrast (DIC) images of FRET1-MC8 cells at P27. (B) Phase-contrast images of migrating and passaged cells isolated from calvaria of FRET mice. (C) Cumulative proliferation rates of three successfully established cell lines. Bars = 100 μm.

All isolated cells clearly and stably expressed the FRET-based tension sensor protein. Representative fluorescence images of donor EGFP and acceptor mCherry, along with differential interference contrast (DIC) images of FRET1-MC8 cells at passage 27 (P27) are shown in Fig. 1A. Phase-contrast images of migrating and passaged cells isolated from bone tissues are also shown in Fig. 1B, revealing that cell morphology remained consistent through multiple passages. Consistent with the morphology described in Kodama’s original study, we also examined the area and shape index of cells and nuclei after 24 h of static culture in both MC3T3-E1 and FRET1-MC8 cells. No significant differences were observed between the two lines (Supplementary Fig. S1). Ten cell lines were established, three of which exhibited robust proliferation, whereas the remaining seven ceased to survive around passage 10. The cumulative proliferation rates of the three successfully established cell lines are presented in Fig. 1C. Particularly, FRET1-MC8 cells exhibited a cumulative proliferation rate of approximately 10^35^ at P50. This value exceeds the reported cumulative proliferation rate of MC3T3-E1 cells (10^25^ at P50) as originally described by Kodama et al. [17], with which we share the same culture and calculation methods. FRET1-MC8 cells continued stable proliferation up to at least P85 and were selected for subsequent experiments.

### 3.2 Osteoblast differentiation

**Fig. 2:**
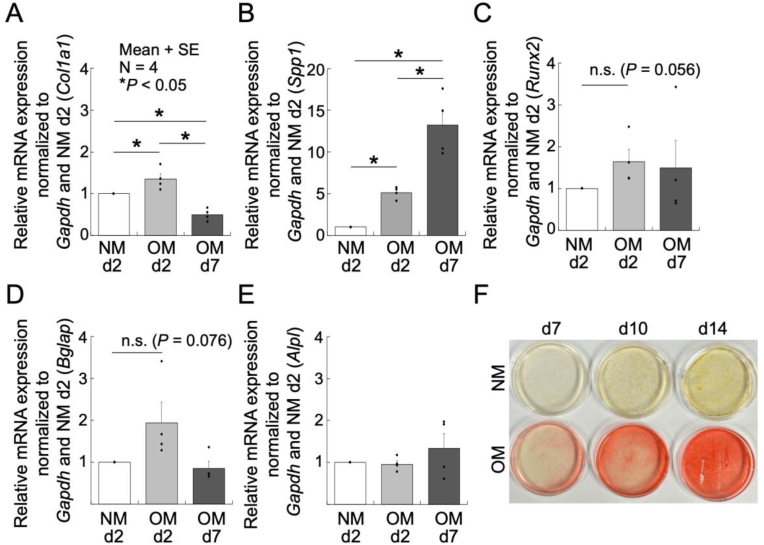
Quantitative real-time PCR analysis of mRNA expression of osteoblastic differentiation genes: *Col1a1* (A), *Spp1* (B), *Runx2* (C), *Bglap* (D) and *Alpl* (E) in FRET1-MC8 cells cultured in normal medium (NM) and osteogenic medium (OM) for 2 and 7 days. (F) Alizarin Red S staining of P65 cells cultured in NM or OM for 7, 10, and 14 days.

As shown in Fig. 2A and B, after 2 days of culture in OM, the expression of osteoblastic differentiation-related genes *Col1a1* and *Spp1* increased by approximately 1.5-fold and 4.5-fold, respectively, compared to NM groups. After 7 days, *Col1a1* expression decreased, while *Spp1* expression further increased more than 2-fold. *Runx2* (Fig. 2C) and *Bglap* (Fig. 2D) expression showed increasing trends under OM conditions, although the differences were not statistically significant. Overall, the established cell line responded to osteogenic culture medium by proceeding toward osteoblastic differentiation.

### 3.3 Evaluation of mineralization capability

Fig. 2F shows Alizarin Red S staining of P65 cells cultured in NM or OM for 7, 10, and 14 days. Cells cultured in NM showed no calcification at all, whereas cells cultured in OM exhibited partial calcification from day 7, becoming extensively calcified by day 14. Importantly, similar results were obtained with cells at earlier passages than P65, which also exhibited extensive calcification after 14 days in OM culture. These results suggest that the established cell line maintains stable mineralization capability over a prolonged culture period.

### 3.4 Evaluation of apparent cellular tension by scratch experiment

**Fig. 3:**
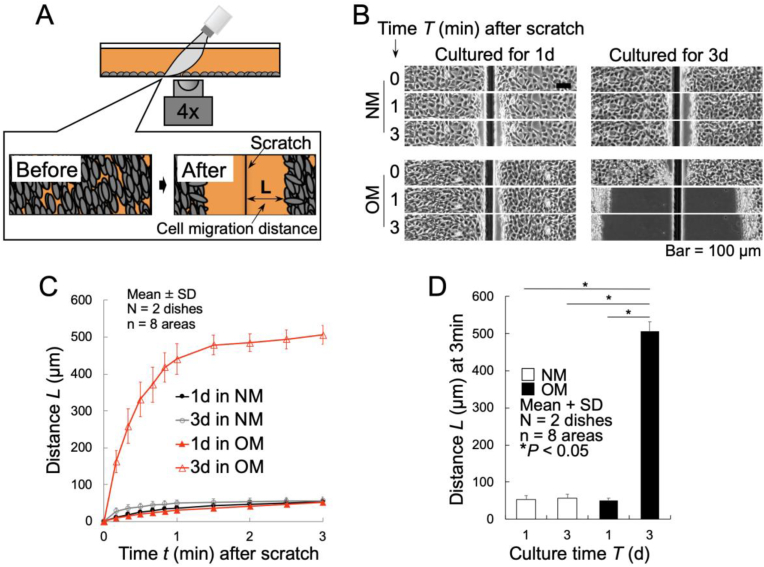
Evaluation of cellular contractility using a scratch experiment. (A) Schematic diagram of the scratch experiment. (B) Representative phase-contrast images of FRET1-MC8 cells at 0, 1, and 3 min post-scratch, cultured in normal medium (NM) and osteogenic medium (OM) for 1 and 3 days. (C) Quantitative analysis of the contraction distance. (D) Total contraction distance over a 3-min post-scratch period.

Representative phase-contrast images at 0-, 1-, and 3-min post-scratch are presented in Fig. 3B. Visual observation indicated minimal cell contraction distances in NM (1d & 3d) and OM (1d) cultures, whereas remarkably larger contraction distances were noted in OM cultures at day 3. Quantitative analysis confirmed this observation (Fig. 3C). Cells rapidly contracted within the first minute, followed by slower movement, with contraction nearly stopping after 1.5 min. The total contraction distance over 3 min was significantly greater in OM-cultured cells at day 3 compared to other groups (Fig. 3D), indicating enhanced cellular tension.

### 3.5 Measurement of intracellular tension by FRET

**Fig. 4:**
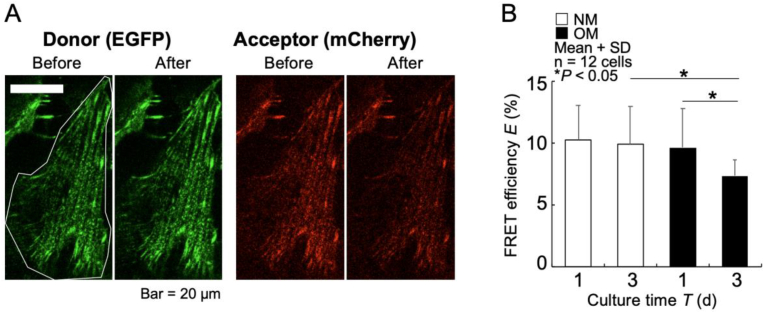
Measurement of intracellular tension changes using FRET efficiency analysis via the acceptor photobleaching method. (A) Donor and acceptor fluorescence images before and after photobleaching of FRET1-MC8 cells cultured in normal medium (NM) for 1 day (white outline). (B) FRET efficiency of FRET1-MC8 cells cultured in NM or osteogenic medium (OM) for 1 and 3 days.

FRET efficiency analysis via the acceptor photobleaching method is shown in Fig. 4. Fig. 4A displays representative donor and acceptor fluorescent images before and after photobleaching of cells cultured in NM for 1 day (white outline). While donor fluorescence intensity increased only slightly, acceptor fluorescence decreased distinctly after photobleaching. Quantitative analysis of the mean FRET efficiency values per cell (Fig. 4B) revealed no significant differences between day 1 and day 3 cultures in NM. However, cells cultured in OM showed approximately 20 % lower FRET efficiency at day 3 compared to day 1, indicating increased intracellular tension.

## 4 Discussion

In the present study, we successfully established a novel osteoblast-like cell line (FRET1-MC8) derived from skull bones of transgenic mice ubiquitously expressing a FRET-based tension sensor [16]. The established FRET1-MC8 cells exhibited a higher proliferative capacity compared to the well-established osteoblast-like cell line MC3T3-E1 (Fig. 1C). Additionally, these cells maintained stable proliferation capability up to at least P85, highlighting their suitability for long-term studies investigating osteoblast differentiation and mineralization processes.

Moreover, the established cells responded effectively to OM by increasing the gene expression of osteoblast differentiation markers *Col1a1* and *Spp1* (Fig. 2A and B). Alizarin Red S staining confirmed mineralization as early as day 7, reaching clear, extensive mineralization by day 14, which is comparable to MC3T3-E1 cells, typically ranging between 12 and 21 days [[24], [25], [26]]. Notably, MC3T3-E1 cells have been reported to lose their mineralization capacity after passage 30 [27], whereas our established cells retained stable mineralization potential even at passage 65. Thus, FRET1-MC8 cells is a highly valuable model for long-term cellular studies of bone remodeling.

In addition, scratch experiments demonstrated an enhanced contractility in cells cultured for 3 days in OM compared to those cultured under other conditions (Fig. 3). However, it remained unclear whether this enhanced contraction was due to increased intracellular tension or reduced cell adhesion. Thus, intracellular tension was quantitatively assessed using the acceptor photobleaching method. While no significant changes were observed in cells cultured in NM, cells cultured in OM for 3 days displayed significantly reduced FRET efficiency, indicating an actual increase in intracellular tension (Fig. 4B). These findings strongly suggest a critical role for increased intracellular tension during osteoblast differentiation and mineralization. Importantly, the simultaneous increase in intracellular tension and *Spp1* expression after day 3 suggests that elevated tension might promote or maintain the expression level of osteogenic genes such as *Spp1*. This hypothesis aligns with previous reports indicating that cytoskeletal structure and nuclear mechanical environment directly influence gene expression and cellular function in osteoblasts [28].

The FRET-based tension sensor used in this study provides a non-invasive method for real-time and quantitative measurement of changes in intracellular tension at specific subcellular locations, offering substantial advantages over conventional techniques such as atomic force microscopy. Our established cell line exhibits stable expression of the tension sensor protein even during extended subculture, making it particularly advantageous for investigating long-term cellular differentiation events such as mineralization. Additionally, this approach allows real-time monitoring of cytoskeletal and tension dynamics without the need for costly reagents, further emphasizing its utility as a powerful experimental platform to elucidate the role of intracellular tension in bone remodeling.

The changes in the expression of *Runx2* and *Bglap* genes were not statistically significant, which may be attributed to the specific timing of their expression during osteoblast differentiation [29]. Monitoring expression levels at additional time points, such as earlier or later stages, and increasing the sample size could provide a more comprehensive understanding of their expression dynamics throughout the differentiation process. Similarly, the lack of increased *Alpl* gene expression under osteogenic induction might reflect suppression effects associated with high concentrations of calcium or phosphate ions [30]. Thus, optimization of culture conditions could potentially enhance *Alpl* expression.

Although real-time live-cell FRET ratio imaging is technically feasible with the established FRET1-MC8 cells, we adopted the acceptor photobleaching method in this study due to the hardware burden of long-term imaging and practical considerations such as focus drift. Although the acceptor photobleaching method is suitable for fixed samples, formalin fixation precludes real-time measurement of dynamic tension changes immediately after stimulation. Future study should incorporate live-cell FRET ratio measurements [15] to provide detailed insights into short-term dynamic changes in tension. Additionally, the 543-nm laser used for photobleaching is suboptimal for exciting mCherry, whose excitation peak is at 578 nm, potentially leading to an underestimation of actual FRET efficiency. Addressing these technical limitations will enhance the accuracy and precision of intracellular tension measurements in future research.

Cell adhesion stability on culture substrates posed another challenge, as cells cultured in OM for more than 7 days detached from glass substrates. Future investigation is needed to identify substrates or culture conditions capable of providing stronger, more stable adhesion for long-term experiments.

Another limitation of the present study is that we did not investigate the genomic stability or the mechanism of immortalization of the FRET1-MC8 cells in detail, such as karyotype analysis or expression of senescence- and DNA damage-related markers (e.g., CDKN2A, TERT, γ-H2AX). Future studies will be needed to confirm the genetic integrity of the cells, particularly given the potential risks of chromosomal changes during long-term culture. In addition, while we analyzed expression of several osteogenic markers, we did not include other important markers such as Ibsp, Sp7 (Osterix), and Dlx5, nor did we perform BMP2 induction experiments. These will be important directions for future work to more comprehensively characterize the differentiation capacity of the established cell line.

In conclusion, the newly established osteoblast-like cell line represents a valuable tool for evaluating mechanical responses during osteoblast differentiation, potentially serving as a foundational technology for advancing research in bone-related diseases and regenerative medicine.

## CRediT authorship contribution statement

**Junfeng Wang:** Writing – original draft, Methodology, Investigation. **Jeonghyun Kim:** Writing – review & editing, Methodology. **Eijiro Maeda:** Writing – review & editing, Methodology. **Takeo Matsumoto:** Writing – review & editing, Supervision, Conceptualization.

## Declaration of competing interest

The authors declare that they have no known competing financial interests or personal relationships that could have appeared to influence the work reported in this paper.

## Data Availability

Data will be made available on request.
