# Supplementary material for: An osteoblast-like cell line derived from mice expressing FRET-based tension sensor reveals cellular tension increase during osteogenic differentiation
Source: Biochem Biophys Rep. 2025 Jul 2;43:102131. doi: 10.1016/j.bbrep.2025.102131 (PMC12270729; doi:10.1016/j.bbrep.2025.102131)
Supplement: Multimedia component 1 [file mmc1.docx]

**Supplementary Information**

**An osteoblast-like cell line derived from mice expressing FRET-based tension sensor reveals cellular tension increase during osteogenic differentiation**

Junfeng Wang, Jeonghyun Kim, Eijiro Maeda, Takeo Matsumoto^*^

Biomechanics Laboratory, Department of Mechanical Systems Engineering, Graduate School of Engineering, Nagoya University, Nagoya, Japan

*Corresponding author

Takeo Matsumoto, Ph.D., Professor

Biomechanics Laboratory

Department of Mechanical Systems Engineering, Graduate School of Engineering

Nagoya University

Furo-cho, Chikusa-ku, Nagoya, Aichi 464-8603, Japan

takeo@nagoya-u.jp

+81-(0)52-789-2721

Supplementary Figure S1


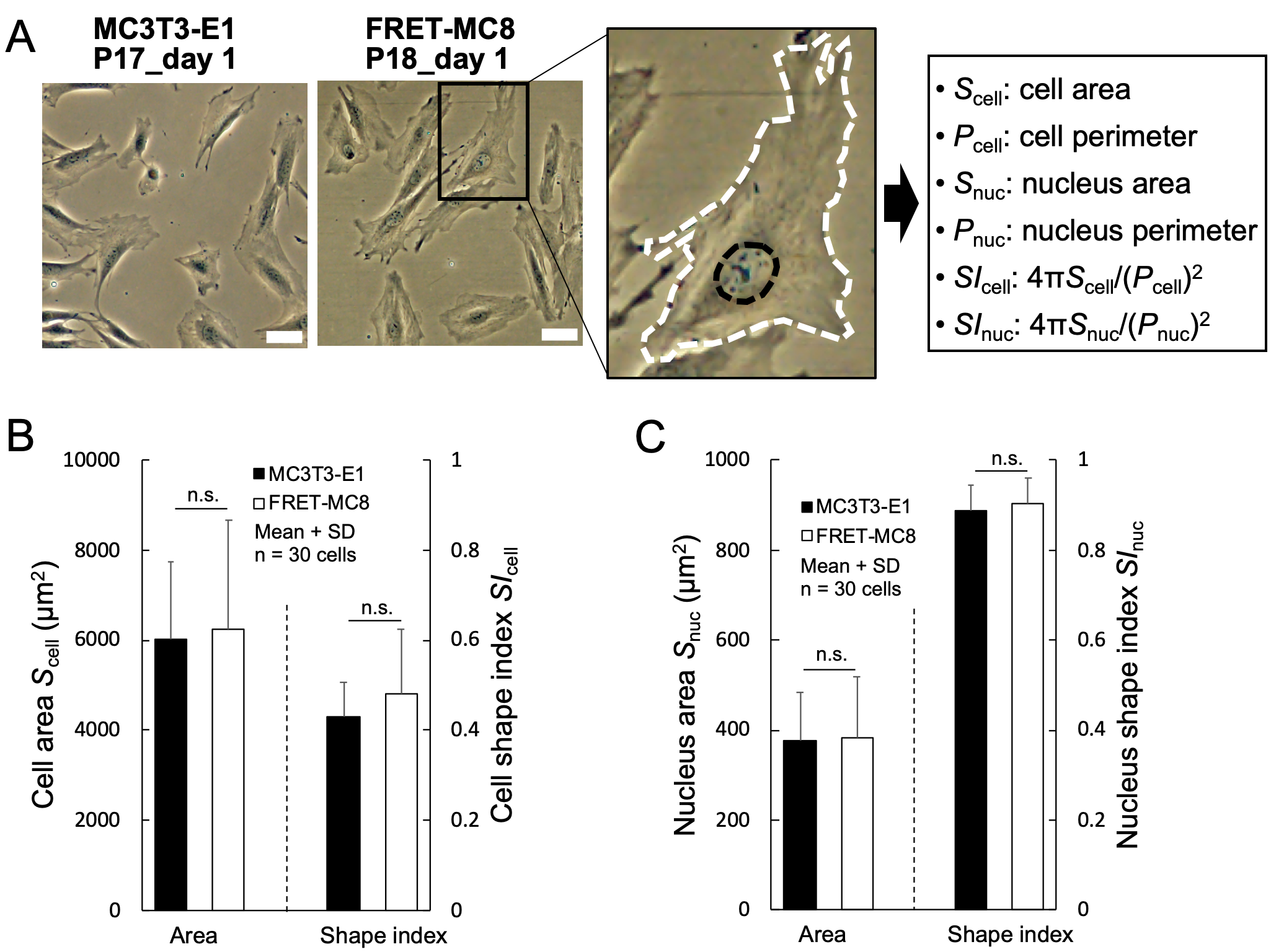


Morphological comparison of cell and nuclear shapes between MC3T3-E1 and FRET1-MC8 cells.

(A) Representative phase-contrast images and definitions of morphological parameters. White dashed lines indicate cell outlines; black dashed lines indicate nuclear outlines. (B) Quantitative analysis of cell area and shape index. (C) Quantitative analysis of nuclear area and shape index. Bars = 100 µm.

Materials and methods

MC3T3-E1 cells (RCB1126, RIKEN BioResource Research Center, Japan) and FRET1-MC8 cells were seeded onto plastic culture dishes and incubated for 24 hours. Phase-contrast images were then acquired using an inverted microscope IX73 (Olympus) equipped with a 10× objective lens (N.A. = 0.3, Olympus)., and cell and nuclear areas (S) and perimeters (P) were measured using ImageJ (version 1.54f, NIH). The shape index was calculated as 4πS/P², where a value closer to 1 indicates a more circular shape, and a value approaching 0 indicates a more elongated or linear shape.
